# A Novel IoT-Enabled Healthcare Monitoring Framework and Improved Grey Wolf Optimization Algorithm-Based Deep Convolution Neural Network Model for Early Diagnosis of Lung Cancer

**DOI:** 10.3390/s23062932

**Published:** 2023-03-08

**Authors:** Reyazur Rashid Irshad, Shahid Hussain, Shahab Saquib Sohail, Abu Sarwar Zamani, Dag Øivind Madsen, Ahmed Abdu Alattab, Abdallah Ahmed Alzupair Ahmed, Khalid Ahmed Abdallah Norain, Omar Ali Saleh Alsaiari

**Affiliations:** 1 Department of Computer Science, College of Science and Arts, Najran University, Sharurah 68341, Saudi Arabia; rrirshad@nu.edu.sa (R.R.I.); aaalattab@nu.edu.sa (A.A.A.); aaahmeed@nu.edu.sa (A.A.A.A.); kanorain@nu.edu.sa (K.A.A.N.); oaalsaiari@nu.edu.sa (O.A.S.A.); 2 Department of Computer Science and Engineering, Sejong University, Seoul 30019, Republic of Korea; shahid.hussain@universityofgalway.ie; 3 Department of Computer Science and Engineering, School of Engineering Sciences and Technology, Jamia Hamdard, New Delhi 110062, India; 4 Department of Computer and Self Development, Preparatory Year Deanship, Prince Sattam bin Abdulaziz University, Al-Kharj 11942, Saudi Arabia; a.zamani@psau.edu.sa; 5 USN School of Business, University of South-Eastern Norway, 3511 Hønefoss, Norway; 6 Department of Computer Science, Faculty of Computer Science and Information Systems, Thamar University, Thamar 87246, Yemen

**Keywords:** Internet-of-Things, healthcare monitoring, lung cancer, tasmanian devil optimization, improved grey wolf optimization, deep convolutional neural network

## Abstract

Lung cancer is a high-risk disease that causes mortality worldwide; nevertheless, lung nodules are the main manifestation that can help to diagnose lung cancer at an early stage, lowering the workload of radiologists and boosting the rate of diagnosis. Artificial intelligence-based neural networks are promising technologies for automatically detecting lung nodules employing patient monitoring data acquired from sensor technology through an Internet-of-Things (IoT)-based patient monitoring system. However, the standard neural networks rely on manually acquired features, which reduces the effectiveness of detection. In this paper, we provide a novel IoT-enabled healthcare monitoring platform and an improved grey-wolf optimization (IGWO)-based deep convulution neural network (DCNN) model for lung cancer detection. The Tasmanian Devil Optimization (TDO) algorithm is utilized to select the most pertinent features for diagnosing lung nodules, and the convergence rate of the standard grey wolf optimization (GWO) algorithm is modified, resulting in an improved GWO algorithm. Consequently, an IGWO-based DCNN is trained on the optimal features obtained from the IoT platform, and the findings are saved in the cloud for the doctor’s judgment. The model is built on an Android platform with DCNN-enabled Python libraries, and the findings are evaluated against cutting-edge lung cancer detection models.

## 1. Introduction

Lung cancer is one of the most common diseases in the world, accounting for 1.3 million diagnoses and 1.2 million deaths per year [1]. The most prevalent lung disorders have been lung cancer, asthma, pneumonia, and pulmonary edema. The respiratory illnesses include lung circulation, tissue disorders, and airway disorders, with asthma being a specific chronic lung disease that worsens the lung’s breathing difficulties [2]. Bronchitis, pneumonia, and bronchiolitis are the three forms of lung infection caused by bacteria and viruses that seriously infect the lungs. They are all harmful to the lungs and can be life-threatening. It permits the oxygen to deliver carbon dioxide, while the humidity level and temperature allow the waste gases to be removed. Incurable lung disorders worsen breathing conditions and leave scars on the body, reducing people’s life expectancy by 25% in five years. The primary activities of the respiratory system are protection, gas exchange, air movement, and sound generation, with the avoidance of lung illnesses and other physical treatments being one of the most important needs for a longer life [3].

Since lung cancer patients do not have symptoms until the disease has advanced to an inoperable level, their chance of survival is just about 15%, even with therapy; however, if diagnosed early, the treatment is highly effective, improving the survival rate by up to 67% [4]. Consequently, one of the main challenges is to come up with strategies that aid in early lung cancer diagnosis and thereby enhance the healthcare system for lung cancer patients [5]. Several procedures, such as chest radiography, sputum cytology, light-induced fluorescence endoscopy, and serum biomarkers, are being used to identify lung cancer, however, none of these have been demonstrated to be effective in accurately diagnosing the disease. Serum tumor indicators on pathological types, staging, monitoring, and prognostication of lung cancer [6] with serum squamous cell carcinoma antigen (SCC) [7], carcinoembryonic antigen (CEA) [8], and neuron-specific enolase (NSE) [9] are regarded to be successful strategies for lung cancer detection; nevertheless, these are laborious techniques [10]. Recent advances in artificial intelligence technology, on the other hand, have resulted in strong neural network models that resemble the human brain system and have shown the ability to anticipate a very complicated system with high accuracy, with little effort and time [11]. On the other hand, the proliferation of sensing technology through the Internet-of-Things (IoT) platform represents sensible objects such as processing capabilities, locating systems, programs, and other devices that sense and acquire data for various reasons [12].

There are three categories of IoT [13]: industrial, consumer, and enterprise IoT, where the manufacturing process is enhanced to employ selections and devices. It links a wide range of products to exchange and link data with smartphones, appliances, and wearables devices. It serves the demands of the consumer and expands the network of interconnected devices, including those connected to thermostats, vehicles, appliances, and homes. The IoT includes devices such as wearables, security systems, trackers, and door locks that capture and exchange data (e.g., temperature, pressure, motion, and light, etc.) to interact with other information across sensors, bridges that perform specifications, activation, security, communication, and detecting activities. It uses sensors to perceive multiple objectives in several domains and has proven efficient in production, agility, mobility, security, and healthcare systems with low costs. However, IoT-based smart healthcare systems face several challenges, including connectivity constraints, system integration complexity, data heterogeneity, security vulnerabilities, privacy risks, and noisy or unreliable sensor data, which can negatively impact overall system performance and reliability [14].

Primary, secondary, and territorial healthcare systems [15] assist people by providing care and sickness prevention. The emergence of IoT technologies enables wearable devices to monitor patients, which are used to diagnose sickness and chronic diseases, thereby improving people’s health and viability, resulting in smart healthcare [16]. The advantages of smart healthcare, which monitors, analyzes, and records health information, include education, immunization, child healthcare, and nutrition. The benefits of smart healthcare services, therefore, include healthcare prevention, rehabilitation, promotion, treatment, and diagnostics. All sensors and devices are connected to specialized devices that communicate with the patient’s body via smart sensors and connected devices. It analyzes the state of healthcare records and provides the information needed to follow patients and their healthcare and access their medical records. The software packages are utilized to get healthcare feedback, knowledge-based decision support, and data from the patient’s respiratory system. Their details are then saved in a database for future planning and training to encapsulate the desired healthcare through healthcare applications. Consequently, it enhances integrated medical care with patient mobility, improved treatment, and cost savings. However, one of the most challenging tasks is acquiring data and identifying the most important attributes, as intimately linked data and its features play a vital role in enhancing the accuracy and sensitivity of these models [17]. In this paper, we developed a novel IoT-enabled healthcare monitoring platform and a grey-world optimization-based deep convolution neural network model with optimal feature selection based on the Tasmanian Devil Optimization algorithm to aid in lung cancer identification. Our contribution is threefold, as detailed below.

1. We developed an IoT platform, inferred the mechanism for acquiring lung disease data, and investigated the process for extracting the most significant attributes employing the Tasmanian Devil Optimization (TDO) algorithm, which enables high accuracy in the diagnosis of lung cancer.
2. We investigate the mechanism of the Grey-Wolf Optimization algorithm and modify its convergence rates, resulting in an improved GWO algorithm that is employed to fine-tune the parameters of the deep convolutional neural network model. Eventually, we presented an IoT-enabled platform with an IGWO-based DCNN model for lung cancer detection.
3. The developed model was trained and tested on the benchmark Exasens dataset, and its accuracy, sensitivity, specificity, and precision were evaluated against state-of-the-art clinical decision support systems (CDSS), regional-based convolutional neural networks (RCNN), active contour method (ACM), and Mask Region-Convolutional Neural Networks (Mask R-CNN) models for lung cancer detection.

The remainder of the article is organized as follows: Section 2 describes the related works. The suggested framework is designed in Section 3, and the experimental findings are discussed in Section 4. Section 5 concludes the article.

## 2. Literature Survey

Almezhghwi et al. [18] introduced a support vector machine, Alex Net, and VGG-16-based deep learning models for image classification of chest X-rays. Their research shows that the first method (Support vector machine) accurately predicts the classification of X-ray image data, while the second and third methods estimate the classification and features of skin lesions. This implies that the multi-class support vector machine uses twelve throat disorders for feature classification, with a robust, rapid, and easy prediction of lung disease.

A novel medical prediagnostic approach combining deep learning and fine-tuning has been shown by Han et al. [19]. Hemorrhagic stroke and lung images are segmented and categorized for classification reasons. The segmentation process classifies the actual images based on the network object of the image. The performance of the suggested approach is measured in terms of accuracy, speed, and efficiency. As a result, several aspects of diseases in the health-of-things domain are examined to refine the classification and segmentation process.

A clinical decision support system (CDSS) was developed by Rehm et al. [20] to identify patient ventilation management in the critical care unit. The IoT device used to transmit and store data from numerous devices and ventilators is monitored by the critical care unit. The development of a machine learning classifier leverages an acute respiratory distress syndrome (ARDS) to anticipate the condition. It improves illness identification, management, and diagnosis. The analyzed scalability problems are optimized, though.

Ahmed et al. [21] used a regional-based convolutional neural network (RCNN) in an IoT-based framework to detect COVID-19. In their model, a deep learning architecture was used to assist COVID-19 identification from samples of chest X-rays amplified by sensors, while the model of the Region Proposal Network (RPN) is used for executing regions and proposals. Distinct datasets are investigated and assessed using a varied learning technique, resulting in accurate and perfect case recognition; as a consequence, diverse images are anticipated for different architectures.

Ma et al. [22] introduced length-of-stay (LOS) to assess paediatric and respiratory disorders using the decision tree method. In their approach, the two techniques of expansion and computation turn dates, texts, and numbers into numeric data from a wide range of data types. Following that, the data is analyzed to construct and test the decision tree algorithms. It accurately classified different ailments while cutting healthcare costs. Therefore, they used multi-class-based classification algorithms to categorize the diseases.

A fully automated approach based on the health of things was demonstrated by Xu et al. [23] to classify CT images. In their model, a two-step technique is employed to classify and segment CT (computed tomography) images, and so the images are classified by 14 models in the first stage by segmenting the lesion into 8 models to discover the existent regions. The image classification approach outperforms in terms of being more precise, reliable, and effective.

To segment CT images of the lungs, Skourt et al. [24] developed a deep learning-based method. In this approach, the segmentation process classifies the image to recover and extract the segmented maps, while the pooling and up-sampling are the two layers used to minimize the spatial dimension of encoder and decoder objects. Although diverse segmentation tasks are conducted in wide regions, they have not explored the classification of lung cancer.

In contrast to the typical method, which complements existing training data to identify the region of lung segments, Medeiros et al. [25] suggested an Active Contour Method (ACM) approach for lung segmentation. In their method, the margins of pulmonary regions increase the fuzzy border detector to better curve adaption, thereby accelerating the region to initialize the lung segments. Although the model appears to perform fast, precisely, and sensitively at the selected level, the system lack to support the real-time diagnostic systems.

Cai et al. [26] devised a Mask Region-Convolutional Neural Network (Mask R-CNN) and ray-casting volume rendering algorithm to undertake the 3D modules of the pulmonary nodule. Their proposed method, which comprises multiple modules such as pre-processing, segmentation, and three-dimensional, was capable of detecting other diseases and improving the segmentation network. The findings demonstrate the applicability of a multi-view technique for various parameters, allowing for more exact segmentation and detection of the pulmonary nodules. Table 1 tabulated the literature survey.

## 3. The Proposed IoT-Enabled Platform with IGWO-Based DCNN Model

**Figure 1:**
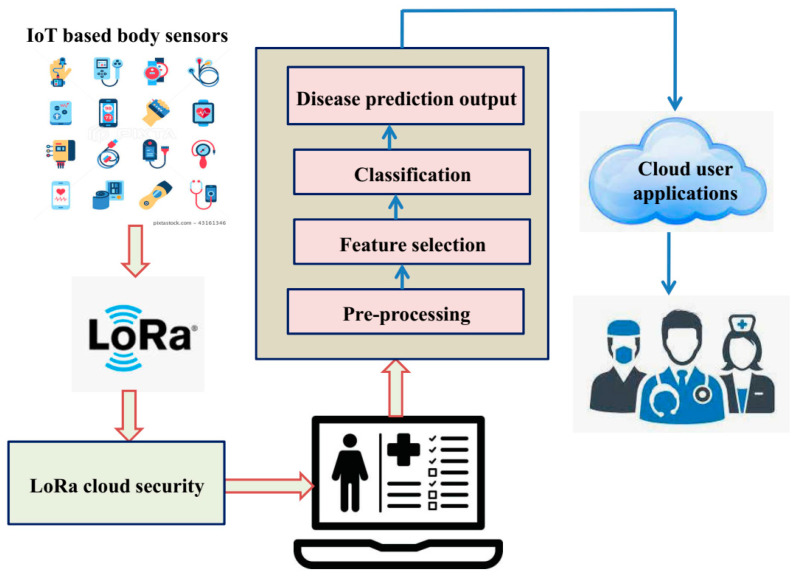
The schematic diagram with workflow of the proposed IoT enabled grey-wolf optimization-based deep convolution neural network (IGWO-based DCNN) model.

The deep CNN accomplishes lung disease classification employing both the training and testing mechanisms, as shown in the block diagram in Figure 1. The figure depicts the IoT body sensor devices that collect each patient’s health data and identify lung cancer using the proposed IoT-enabled IGOW-based DCNN model representing a detailed healthcare monitoring system, and is explained in the following sub-section.

### 3.1. Pre-Processing

Pre-processing is important in rationalizing the data for effective algorithm use and thus helps to clean the dataset by removing redundancies and dealing with missing values. Consequently, we verified the patient’s blood pressure, cholesterol levels, and age groups, and replaced the missing attribute with specified values [27], such that the values are substituted in the same position for the matched attributes [28]. Furthermore, the patient’s health concerns are classified into distinct groups based on the type of lung pain.

### 3.2. Feature Selection

Considering the hunting behavior of the Tasmanian devil, which alternates between scavenging (carrion feeding) and active hunting, the Tasmanian Devil Optimization (TDO) algorithm models exploration and exploitation phases to search for optimal solutions. This bio-inspired strategy enables effective feature selection by balancing global exploration and local exploitation of the search space [29]. The features’ optimal control mechanism imitates the Tasmanian devil’s algorithm search and attempts to locate the most suitable food sources as given by Equation (1).

(1) $$MA={\left[\begin{array}{c} MA_{1}\\ \vdots \\ MA_{j}\\ \vdots \\ MA_{X} \end{array}\right]}_{X\times Y}=\;{\left[\begin{array}{ccccc} ma_{1,1} & \cdots & ma_{1,k} & \cdots & ma_{1,y}\\ \vdots & \ddots & \vdots & ⋰ & \vdots \\ ma_{1,1} & \cdots & ma_{j,k} & \cdots & ma_{j,y}\\ \vdots & ⋰ & \vdots & \ddots & \vdots \\ ma_{1,1} & \cdots & ma_{x,k} & \cdots & ma_{x,y} \end{array}\right]}_{X\times Y}$$

The preliminary Tasmanian devil population is signified as *MA*, and the $j^{th}$ potential solution is signified as $Ma_{j}$ . Likewise, the applicant valuation of the $k^{th}$ value is defined as $Ma_{j,k}$ , and the space, including the problems and factors y , is symbolized as X . The objective function is expressed in the following Equation (2).

(2) $$Fit={\left[\begin{array}{c} Fit_{1}\\ \vdots \\ Fit_{j}\\ \vdots \\ Fit_{X} \end{array}\right]}_{X\times 1}=\;{\left[\begin{array}{c} Fit\left(Ma_{1}\right)\\ \vdots \\ Fit\left(Ma_{j}\right)\\ \vdots \\ Fit\left(Ma_{X}\right) \end{array}\right]}_{X\times 1}$$

where F represents the optimal solution and $Fit_{j}$ represents the $j^{th}$ candidate solution of the objective function.

**Phase of exploration:** The Tasmanian devil in the $j^{th}$ position chooses carrion first from the neighborhood by estimation based on the choice of random conditions as expressed in Equation (3).

(3) $$DB_{j}=Ma_{i},\;\;i\;\in \;\left\{1,\;2,\;\cdots ,\;X\left|i\neq j\right.\right\},\;j=1,2,\cdots ,X$$

The chosen carrion in the $j^{th}$ Tasmanian devil is depicted by $DB_{j}$ . The new place is dependent on the chosen carcasses in the search space. The new agent’s updated place could be formulated as given in Equations (4) and (5).

(4) maj,knew, R1=maj,k+S.Dj, k−I.mj,k,        Gdj<Gj maj,k+S.Dj, k−mj,k,        Otherwise

(5) Maj=maj,knew, R1,  Gjnew, R1<Gj Maj,       Otherwise

Based on the initial strategic plan, the new and updated condition has been signified as $ma_{j,k}^{new,R_{1}}$ . The absolute function and the arbitrary durations are both between 0 and 1, while the random number I is between 1 and 2.

**Phase of exploitation:** Depending on the position of the predators, the same location of other Tasmanian devil populations can be assumed with the choice of prey as described by Equation (6).

(6) $$P_{j}=Ma_{i},\;\;i\;\in \;\left\{1,\;2,\;\cdots ,\;X\left|i\neq j\right.\right\},\;j=1,2,\cdots ,X$$

When it discovers an improved result based on the objective function, it changes its current stance and decides on a new role as expressed by Equations (7) and (8).

(7) maj,knew, R2=maj,k+S.aj, k−I.mj,k,        Gqj<Gj maj,k+S.mj, k−aj,k,        Otherwise

(8) Mj=Mj,knew, R2,  Gjnew, R2<Gj Mj,       Otherwise

Considering the Devil chosen value as a target of predators and following the previous value, an optimal scheduling solution is established, and the procreation of the devil’s place is approximated following Equations (9)–(11).

(9) $$ma_{j,k}^{new}=ma_{j,\;k}+\left(2s-1\right).s.ma_{j,\;k}\;\;$$

(10) $$s=0.01\;\left(1-\frac{1}{maximum\;iteration}\right)\;\;$$

(11) Maj=Majnew,  Gjnew<Gj Maj,       Otherwise

The current incarnation is portrayed as an *iteration*, while the total number of iterations is indicated as the *maximum iteration*. The most pertinent and low-dimensionality features are picked from the dataset attributes as a result of its new status, where the *j^th^* Tasmanian devil of the neighbourhood is $M_{j}^{new}$ and the fitness value is $G_{j}$ .

### 3.3. Improved Grey Wolf Optimization Algorithm-Based Deep-CNN for Lung Cancer Detection

The IoT-enabled healthcare monitoring system allows the Improved Grey Wolf Optimization (IGWO) algorithm to fine-tune parameters for training via the Deep Convolutional Neural Network (DCNN) model, resulting in high detection efficiency of lung cancer. This section explores the IGWO’s operating mechanism for parameter tuning and the IGWO-based DCNN model.

- Improved Grey Wolf Optimization

The social leadership hierarchy and group hunting behaviour of GWO are improved in terms of convergence rate, resulting in the IGWO that determines the optimal solution in group β being placed at the top of the hierarchical pyramid to assist the rest of the group and the position of the prey as presented in Equation (12). The IGWO is then used to integrate data from the IoT-enabled healthcare system to fine-tune the settings for training through the DCNN for lung disease diagnosis.

(12) $$M_{r}^{k\left(t\right)}=\left(\left(W_{\beta }\times M_{\beta }^{k}\left(t\right)\right)+\left(W_{\gamma }\times M_{\gamma }^{k}\left(t\right)\right)+\left(W_{\delta }\times M_{\delta }^{k}\left(t\right)\right)\right)+\mu \left(t\right)\;\;$$

The solution to each d dimension issue can be represented as k=1, 2,⋯,d and the position of the prey can be evaluated for the $k^{th}$ element at the t iteration and is represented as $F:M_{r}^{k}\left(t\right)$ . The rigorous social leadership of the grey wolf pack and its weights can be represented as $W=\left(W_{\beta },W_{\gamma },W_{\delta }\right)$ and each weight should be between 0 and 1 such that their aggregate equals 1, as indicated by the inequality and equity criteria provided in Equations (13) and (14), respectively [30].

(13) $$1\geq \;W_{\beta }>W_{\gamma }>W_{\delta }\geq 0\;$$

(14) $$W_{\beta }+W_{\gamma }+W_{\delta }=1$$

The stochastic error can be evaluated as $\mu \left(t\right)\sim X\left(0,\;\sigma \left(t\right)\right)$ [31], where the standard deviation and mean of the Gaussian distribution are stated as $\sigma \left(t\right)$ and $\mu \left(t\right)$ . Consequently, we define the property of dynamic deviation as given in Equation (15) and thereby we update the location of the $k^{th}$ wolf as represented by Equation (16). Eventually, at the $k^{th}$ dimension at the $t^{th}$ and $j^{th}$ solutions are regarded as $M_{j\left(t\right)}^{k}$ .

(15) $$\sigma \left(t\right)>\sigma \left(t+1\right)$$

(16) $$M_{r}^{k}\left(t+1\right)=M_{r}^{k}\left(t\right)-R\times \left|M_{r}^{k}\left(t\right)-M_{j\left(t\right)}^{k}\right|$$

The new local optimal is explored provided that $\left|R\right|>1$ by employing the random (*R*) in the interval [−2, 2] and the prey searching and attacking are implied utilizing the $\left|R\right|>1$ and $\left|R\right|<1$ conditions, respectively. The new position can be derived using Equation (5) outside the restriction, where the constraints are estimated by random steps, as shown in Equation (17).

**Figure 2:**
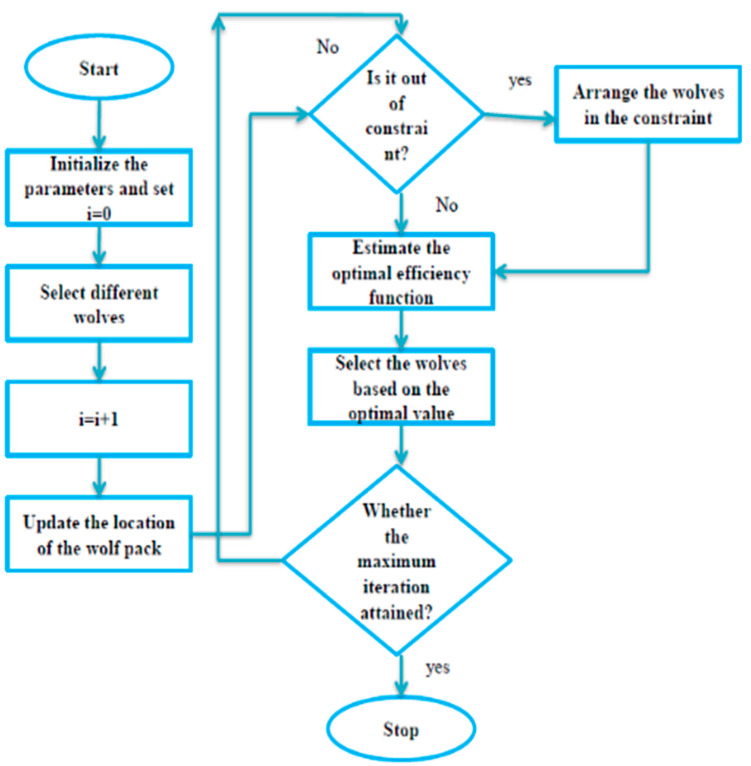
Systematic flowchart of the Grey Wolf Optimization algorithm for fine-tuning of the parameters.

(17) Mrkt+1=Mrkt+v×Uk−Mjkt,   if Mjkt+1>Uk Mrkt+v×Lk−Mjkt,   if Mjkt+1>Lk

where $U^{k}$ and $L^{k}$ indicate the upper and lower boundaries of the constraints, respectively, and V is an arbitrary value between 0 and 1. The wolf’s random movements are considerably determined when executing the prey-finding process, as shown in the systematic flowchart of the standard GWO algorithm in Figure 2.

Given that the GWO converges slowly, with a lower convergence rate at the early stage and a higher convergence rate at the latter stage [32,33], we updated the convergence rates at the early and later stages by integrating Equations (18) and (19), respectively.

(18) $$h=0.9\times \left(2-t\times \frac{2}{Max\_iter}\right)\;\;$$

(19) $$h=1.2\times \left(2-t\times \frac{2}{Max\_iter}\right)\;\;$$

where h is the convergence rate, t is the iteration index, and Max_iter is the total number of iterations. Likewise, to minimize the local optimal fall, we utilize sine-cosine functions illustrated in Equation (20), and the resulting improved GWO algorithm is then employed to fine-tune the parameters for training through the DCNN for lung cancer diagnosis.

(20) $$M_{j}^{k}\left(t+1\right)=\sin\left(t\right)M_{1}+\sin\left(t\right)\cos\left(t\right)M_{2}+\cos\left(t\right)M_{3}$$

B. Improved grey wolf optimization algorithm-based deep convolution neural network model

**Figure 3:**
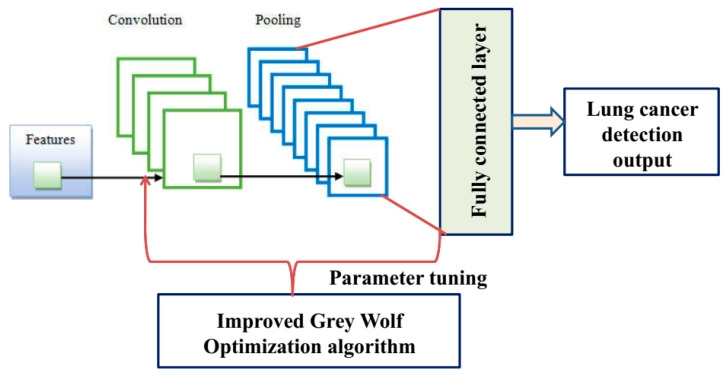
An illustration of integrating the improved grey wolf optimization algorithm to the deep convolution neural network for fine-tuning of the parameters.

The deep convolution neural network classifier configuration comprises several layers, each of which defines a combination of nodes and functionalities, as depicted in Figure 3. The convolution layer generates image features, while the classification strands generate the final output [34]. The DCNN classifier’s input layer is initially applied utilizing feature vectors recorded from the lung cancer patients, and the feature maps are further reduced by employing convolutional filters in the deep convolution neural network model for lung cancer detection. The neurons between layers communicate with each other via configurable heaps with the gradient output, which can be mathematically characterized as demonstrated in Equation (21).

(21) $$CV^{p+1}=CV^{p}+\overset{J}{\underset{f=0}{\sum }}\overset{J}{\underset{h=0}{\sum }}{\left(We\right)}_{f}\times {\left(bi\right)}_{f}\;$$

In the above equation, * denotes the convolution layer and $CV^{p+1}$ signifies the repaired extracted features of the convolution layer, where the weight lifting of the convolution layer identified by ${\left(we\right)}_{f}$ and the bias denoted by ${\left(bi\right)}_{f}$ are optimally optimized utilizing IGWO algorithm [35].

The learning behaviour of the lung cancer detection model is improved by integrating a batch normalization layer between the convolution operation and the ReLU layer; as a result, this layer is accountable for regulating both the gradients and the authorizations in the system to ensure efficient training. The local information is transferred from the convolution layer to the quasi-ReLU layer, which has neither mass nor bias. Obtaining bottom-up samples from the returned features from the convolution layer within the max-pooling enables the reduction in information and geographic size. Depending on the preceding layer of the convolutional layer, the identical process of producing the finished piece within the fully connected layer is represented by Equations (22) and (23).

(22) $$AR=X\left(CV^{p+1}\right)\;$$

(23) $$C^{p+1}=CV^{p}+\overset{J}{\underset{f=0}{\sum }}\overset{J}{\underset{h=0}{\sum }}{\left(CV\right)}_{f}\times {\left(Bias\right)}_{f}$$

This implies that the suggested IGWO algorithm determines the weights of the training DCNN classifier for the optimum solution. Eventually, the output FC layer is regularized using the SoftMax activation function to facilitate efficient classification layer processing [36]. The categorization covering, which is the topmost layer of DCNN, applies the options generated by the SoftMax activation to each and all input data intended to check lung cancer for each selected feature vector [37,38]. Finally, the proposed IGWO-based DCNN model recognizes different stages of lung disorders such as asthma, chronic obstructive pulmonary disease (COPD), and normal.

## 4. Results and Discussion

This section describes the experimental setup, the dataset used, the results obtained, and the comparative outcomes in a broader context. The proposed IoT-based lung disease prediction healthcare monitoring system is built on the Android operating system and Python libraries.

### 4.1. Dataset Explanation

The proposed IGWO-based DCNN model is trained on the Exasens dataset [39], a lung diseases dataset with four types of respiratory diseases: asthma, chronic obstructive pulmonary disease (COPD) infected, and normal. The experimental results are incorporated on an NS-2 GPU-based computer with a16GB RAM and GTX1050 GPU as well as an Intel Core i5-8300H CPU trying to run TensorFlow 1.15. Belong to this dataset, 70% of data for training and rest of 30% data used for testing purpose. The optimized deep learning model is trained by adjusting the network configuration so that the modeling gets better over the course of training. The dataset, additionally, includes demographic data collected from sample saliva of four groups acquired from the Research Center Borstel, Bio Material Bank Nord (Borstel, Germany) following the samples collection regulations of the Luebeck University ethics committee [39].

### 4.2. Experimental Setup

The proposed work has been implemented with the incorporation of the microcontroller and other readily available hardware devices, coupled with the LoRa communication hardware that forwards the data to the cloud storage system. Different features such as the patient’s age, chronic diseases, and gender have been saved and combined with the patient’s ID in the suggested system. The data collection and processing are carried out on a Raspberry Pi single-board computer with the hardware configuration described in Table 2.

### 4.3. Performance Metrics

The proposed IGWO-based DCNN for lung disease is assessed using sensitivity, specificity, accuracy, precision, disease prevalence, and negative predictive value. The outcomes are evaluated against cutting-edge clinical decision support systems (CDSS) [20], regional-based convolutional neural networks (RCNN) [21], active contour method (ACM) [25], and Mask Region-Convolutional Neural Networks (Mask R-CNN) [26]. For clarity, the performance measurements are discussed in more detail below.

(i) Sensitivity

The sensitivity evaluates the various methods by assessing the capacity of the anticipating based on the features acquired against the method’s estimated outcomes, as indicated in Equation (24).

(24) $$Sen=\frac{G_{p}}{G_{p}+H_{f}}\;$$

(ii) Specificity

The specificity describes the ratio of true negative values to the total number of false cases in the system, with values of 0.0 and 1.0 for the worst and best case scenarios, respectively, and numerically can be stated as shown in Equation (25).

(25) $$Spe=\frac{G_{f}}{G_{f}+H_{f}}\;$$

(iii) Accuracy

The accuracy is measured as the proportion of properly classified predictions (points) to the total number of predictions between 0 and 1, and it relies on how the data is normalized for the algorithm, as given in Equation (26).

(26) $$Acc=\frac{G_{p}+G_{f}}{G_{p}+G_{f}+H_{f}+H_{p}}\;$$

(iv) Precision (Negative Predict Value)

The precision measures the number of accurate positive predictions made by the algorithm, and it is determined mathematically as the ratio of properly predicted positive instances divided by the total number of positive examples anticipated, as provided in Equation (27).

(27) $$Pre=\frac{G_{p}}{G_{p}+H_{p}}\;$$

(v) Disease Prevalence

The disease prevalence is the clinical prognosis that defines the likelihood of discovering the disease (i.e., lung disease) among patients before the screening test and can be expressed mathematically, as described in Equation (28).

(28) $$DP=\frac{G_{p}+G_{f}}{G_{p}+G_{f}+H_{f}+H_{p}}\;$$

(vi) Negative Predict Value

The negative predictive value (NPV) is the proportion of patients with negative test results who are already healthy, and it is mathematically defined as the ratio of subjects properly classified as negative to all those with negative test findings (Equation (29)).

(29) $$NPV=\frac{G_{f}}{\;G_{f}+H_{p}}\;$$

In the above Equations (28) and (29), the $G_{p}$ represents the true positive, $G_{f}$ denotes the true negative, $H_{p}$ denotes the false positive, and $H_{f}$ denotes the false negative, respectively.

### 4.4. Comparative Study

**Figure 4:**
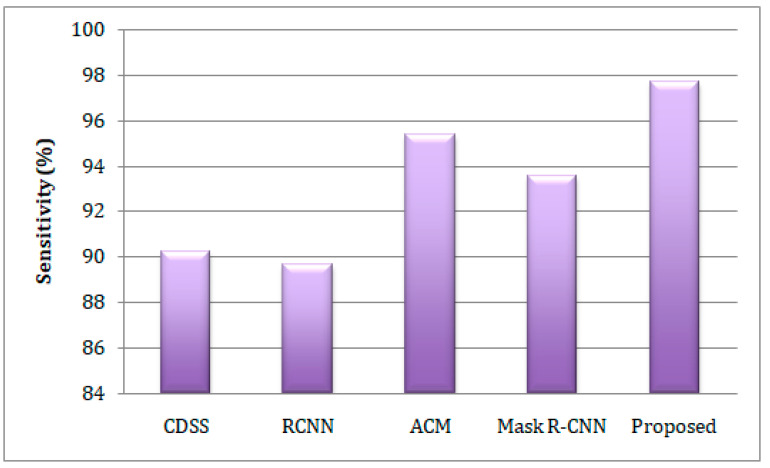
A comparison of the estimation of sensitivity corresponding to the different lung disease predictive models.

**Figure 5:**
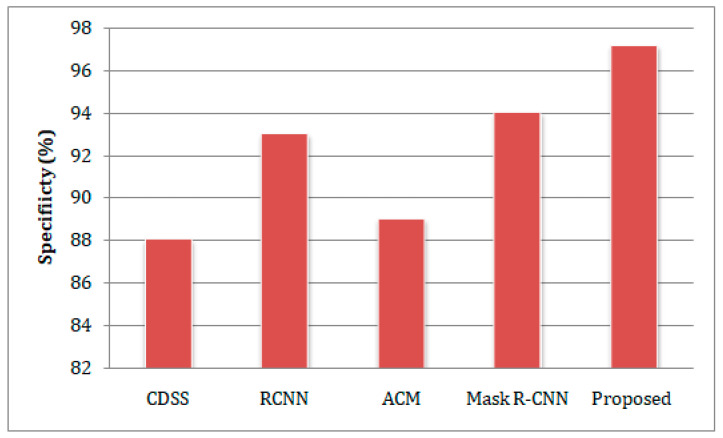
A comparison of the estimation of specificity concerning the different methods.

In this section, we contrast the proposed IGWO-based DCNN model’s performance to that of the existing CDSS [20], RCNN [21], ACM [25], and Mask R-CNN [26] models while taking into account the aforementioned performance assessment criteria. In Figure 4, the sensitivity comparison analysis of the different lung disease-predicting models is presented. The graph indicates that employing the IGWO algorithm enhances the performance of the DCNN by attaining the highest sensitivity of 97.67%. Meanwhile, CDSS [20], RCNN [21], ACM [25], and Mask R-CNN [26] reach 90.21%, 89.67%, 95.34%, and 93.54%, respectively. The work’s specificity is compared to comparable works and plotted in a graphical form, as seen in Figure 5. From the graph, it can be seen that the suggested work has a higher specificity of 98.12%, whereas other works have a lower proportion. The suggested IGWO expands search-ability and, as a result, expands the prediction of lung diseases and reduces inaccurate prediction.

**Figure 6:**
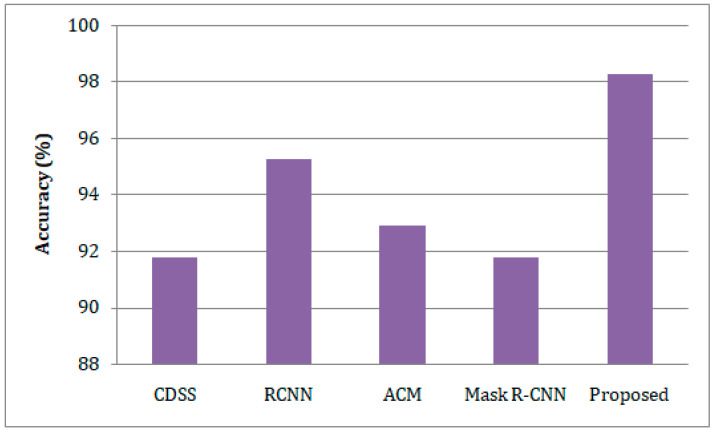
A comparison of the estimation of accuracy for lung disease with respect to the different predictive models.

**Figure 7:**
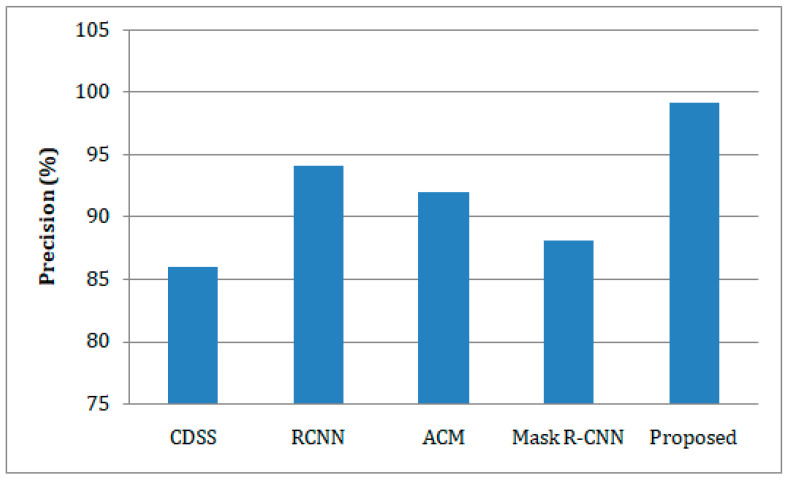
A comparison of the estimation of precision for lung disease with respect to the different predictive models.

In comparison to existing methodologies, the presented study has greater prediction accuracy, as depicted in Figure 6. The lung disease prediction of our suggested healthcare monitoring system reaches 98.27%, whereas the techniques CDSS [20], RCNN [21], ACM [25], and Mask R-CNN [26] show 91.78%, 95.26%, 92.89%, and 91.76%, respectively. Figure 7 shows the precision-based comparison analysis. When compared to the other methods, which only achieve low precision, our suggested strategy obtains a precision of 99.15%. The suggested work was followed by the RCNN and ACM, which achieved around 94.0% and 92.0% precision, respectively, compared to the CDSS and Mask-RCNN, which achieved 88.0% and 88.5% precision, respectively.

The disease prevalence values and prediction values of the suggested and current techniques are compared in Table 3 and Table 4 while taking into account the PPV and NPV values. It should be noted that the highest PPV value and vice versa have been attained when the disease prevalence is higher. In comparison to the DCSS, ACM, RCNN, and Mask-RCNN, the suggested IoT-enabled healthcare system outperforms with higher values of the NPV and PPV. The PPV value of our suggested technique, for instance, is 99.78% when the records are equal to 5000, which indicates that lung disease is likely to occur following the screening test in this case. When evaluating 5000 records, however, the NPV value of our proposed strategy is 99.69%, which is greater than the other alternatives. Computational time analysis is depicted in Table 5. The time of Gray wolf optimization (GSO) is 8.1 s which is minimum to PSO and GA.

## 5. Conclusions

In this study, we introduced a novel IoT-enabled healthcare monitoring platform based on an Improve Grey Wolf Optimization (IGWO)-based deep convolutional neural network (DCNN) model. The fundamental mechanism of the Tasmanian Devil Optimization (TDO) algorithm for identifying the most relevant features for diagnosing lung disease is investigated. The standard GWO’s convergence rate is modified, and the resulting improved GWO algorithm is used to fine-tune the parameters of the DCNN model. The suggested framework is developed using the Python DCNN libraries and the Android operating system, and it is tested by employing the Exasens benchmark dataset from the Research Center Borstel and the Bio Material Bank Nord (Borstel, Germany). The dataset consists of demographic data taken from saliva samples of four different groups, including those with asthma, chronic obstructive pulmonary disease (COPD), infection, and normal. Following the simulation outcomes, the suggested IGWO-based DCNN model outperform to the existing CDSS, RCNN, ACM, and Mask R-CNN models in terms of precision, accuracy, sensitivity, and specificity. In more detail, the suggested approaches have precision, accuracy, sensitivity, and specificity of 99.15%, 98.27%, 97.67%, and 98.12%, which are superior to the current CDSS, RCNN, ACM, and Mask R-CNN methods.

## Figures and Tables

**Table 1:** Literature Survey.

Author	Methods	Advantages	Limitations
Almezhghwi et al. [18]	support vector machine, Alex Net, and VGG-16-based deep learning models	A robust, rapid, and easy prediction of lung disease	Minimum scalability
Han et al. [19]	Medical prediagnostic approach	Higher accuracy, speed, and efficiency	Less aspect of health-of-things domain
Ahmed et al. [21]	RCNN	Resulting in accurate and perfect case recognition	Computational; difficulties
Ma et al. [22]	Length-of-stay (LOS)	Accurately classified different ailments	Huge cost
Xu et al. [23]	A fully automated approach	More precise, reliable, and effective	Not suitable for big dataset
Skourt et al. [24]	Deep learning	Minimize the spatial dimension of encoder and decoder objects	Not explored the classification of lung cancer
Medeiros et al. [25]	Active Contour Method (ACM) approach	Perform fast, precisely, and sensitively	System lack to support the real-time diagnostic systems.
Cai et al. [26]	Mask R-CNN	Capable of detecting other diseases and improving the segmentation network	Higher time taken for execution

**Table 2:** The hardware and their configuration setup used in the experiment.

Hardware	Explanation
SX1272	Act as transmitter and receiver with 900 MHz LoRa
AD8232	The electrocardiographic board used in Analog Devices
User Computer	Inter^®^ Core^TM^ i5-2400CPU@3.10 GHz PC
Raspberry Pi-IV	1.5 GHz quad-core 64-bit ARM Cortex-A72 CPU

**Table 3:** Disease prevalence analysis based on positive predicted values (PPV).

DP	Records	PPV(%)				
		CDSS	RCNN	ACM	Mask R-CNN	Proposed
67	567	86.56	89.90	91.23	94.46	98.87
79	895	92.65	91.67	95.45	93.36	99.23
100	1568	93.56	93.56	92.56	96.78	99.45
198	5000	94.34	94.89	93.66	97.78	99.78

**Table 4:** Disease prevalence analysis based on negative predicted values (NPV).

DP	Records	NPV(%)				
		CDSS	RCNN	ACM	Mask R-CNN	Proposed
67	567	92.45	89.45	90.67	93.56	98.67
79	895	89.45	90.35	91.63	94.29	98.89
100	1568	91.98	91.56	92.40	95.78	99.45
198	5000	94.78	92.11	93.00	96.28	99.69

**Table 5:** Computational time analysis.

Algorithms	Time in Seconds
Particle swarm optimization (PSO)	13 (s)
Genetic Algorithm (GA)	10 (s)
Gray wolf optimization (GSO)	8.1 (s)

## Data Availability

Not applicable.
