# Correction: Irshad et al. A Novel IoT-Enabled Healthcare Monitoring Framework and Improved Grey Wolf Optimization Algorithm-Based Deep Convolution Neural Network Model for Early Diagnosis of Lung Cancer. *Sensors* 2023, *23*, 2932

**DOI:** 10.3390/s26103099

**Published:** 2026-05-14

**Authors:** Reyazur Rashid Irshad, Shahid Hussain, Shahab Saquib Sohail, Abu Sarwar Zamani, Dag Øivind Madsen, Ahmed Abdu Alattab, Abdallah Ahmed Alzupair Ahmed, Khalid Ahmed Abdallah Norain, Omar Ali Saleh Alsaiari

**Affiliations:** 1 Department of Computer Science, College of Science and Arts, Najran University, Sharurah 68341, Saudi Arabia; 2 Department of Computer Science and Engineering, Sejong University, Seoul 30019, Republic of Korea; 3 Department of Computer Science and Engineering, School of Engineering Sciences and Technology, Jamia Hamdard, New Delhi 110062, India; 4 Department of Computer and Self Development, Preparatory Year Deanship, Prince Sattam bin Abdulaziz University, Al-Kharj 11942, Saudi Arabia; 5 USN School of Business, University of South-Eastern Norway, 3511 Hønefoss, Norway; 6 Department of Computer Science, Faculty of Computer Science and Information Systems, Thamar University, Thamar 87246, Yemen

There were errors in the original publication [1]. Some references cited in the feature selection subsection were not directly relevant to the algorithmic description.

**Text Correction**

In Section 1, Paragraph 3, the content reads “The performance of the IoT system is, however, hindered by several limitations, including connectivity challenges, integration issues, complexity, security concerns, privacy concerns, and noisy data [14].” This has been updated to “However, IoT-based smart healthcare systems face several challenges, including connectivity constraints, system integration complexity, data heterogeneity, security vulnerabilities, privacy risks, and noisy or unreliable sensor data, which can negatively impact overall system performance and reliability [14].”

In Section 3.2, Paragraph 1, the content “Considering the Tasmanian devil hunting behaviour patterns, it presents two sources of food, feeding on carrion and hunting and nourish on prey [29], which is employed to accomplish the optimal control, enabling the Tasmanian Devil Optimization (TDO) algorithm to acquire the important features that are closely related to lung cancer diagnosis. ” has been updated to “Considering the hunting behavior of the Tasmanian devil, which alternates between scavenging (carrion feeding) and active hunting, the Tasmanian Devil Optimization (TDO) algorithm models exploration and exploitation phases to search for optimal solutions. This bio-inspired strategy enables effective feature selection by balancing global exploration and local exploitation of the search space [29].”

**References Correction**

References 1, 2 and 14 have been replaced by the following references:

1. Sung, H.; Ferlay, J.; Siegel, R.L.; Laversanne, M.; Soerjomataram, I.; Jemal, A.; Bray, F. Global Cancer Statistics 2020: GLOBOCAN Estimates of Incidence and Mortality Worldwide for 36 Cancers in 185 Countries. *CA Cancer J. Clin.* **2021**, *71*, 209–249.

2. Momtazmanesh, S.; Moghaddam, S.S.; Ghamari, S.H.; Rad, E.M.; Rezaei, N.; Shobeiri, P.; Aali, A.; Abbasi-Kangevari, M.; Ab-basi-Kangevari, Z.; Abdelmasseh, M.; et al. Global burden of chronic respiratory diseases and risk factors, 1990–2019: An update from the Global Burden of Disease Study 2019. *eClinicalMedicine* **2023**, *59*, 101936. https://doi.org/10.1016/j.eclinm.2023.101936.

14. Mehmood, Y.; Ahmad, F.; Yaqoob, I.; Adnane, A.; Imran, M.; Guizani, S. A Comprehensive Survey of the Internet of Things (IoT) and AI-Based Smart Healthcare. *IEEE Access* **2021**, *9*, 3660–3686. 

References 30 and 31 have been removed. With this correction, the order of some references has been adjusted accordingly.

The authors state that the scientific conclusions are unaffected. This correction was approved by the Academic Editor. The original publication has also been updated.
